# Supplementary material for: Neural mechanisms for selectively tuning in to the target speaker in a naturalistic noisy situation
Source: Nat Commun. 2018 Jun 19;9:2405. doi: 10.1038/s41467-018-04819-z (PMC6008393; doi:10.1038/s41467-018-04819-z)
Supplement: Supplementary file 1 — Supplementary Information [file 41467_2018_4819_MOESM1_ESM.pdf]

Neural mechanisms for selectively tuning in to the target speaker in a naturalistic noisy situation

Dai et al.

Supplementary Methods

Assessment of the communication behaviors in the two speakers

Immediately after each task, the listener was asked to assess several aspects of the communication behaviors with each speaker on a 5-point scale (1 represents the lowest level, and 5 represents the highest level). This assessment was based on the whole period of the task and included two aspects, e.g., verbal and non-verbal communications. For verbal communications, 6 items were included: 1) Speed; 2) Loudness; 3) Fluency; 4) Naturalness of intonation; 5) Clarity; and 6) Appropriateness in wording and syntax. Internal consistency across the 6 items was high for both the face-to-face condition (Cronbach's alpha = 0.827 for the speaker on the left side from the listener's perspective and 0.934 for the speaker on the right side) and the back-to-back condition (Cronbach's alpha = 0.893 for the left speaker and 0.910 for the right speaker). The non-verbal aspect also had 6 items: 1) Naturalness; 2) Frequency of nodding; 3) Frequency of hand gestures; 4) Frequency of facial expressions; 5) Frequency of eye gaze; and 6) Frequency of smiling. The inter-item consistency was also satisfactory to high in the face-to-face condition (Cronbach's alpha = 0.8 for the left speaker and 0.851 for the right speaker) and the back-to-back condition (Cronbach's alpha = 0.882 for the left speaker and 0.884 for the right speaker). Thus, scores of items were summed to have an overall score for the patterns of verbal communication and that of non-verbal communication respectively. Results did not show any significant differences between the two speakers (Mann-Whitney *U* test, $P > 0.05$, Šídák correction, see Methods).

Two additional coders who did not take part in this experiment were asked to transcribe the speech of the two speakers based on the experimental video. Based on the transcriptions, speaking rate (i.e., the length of speaking period was divided by the number of Chinese characters uttered in the speech) and percentage of disfluency (i.e., repetitions, pauses, and interjections) were calculated. There were again no significant differences between the attended and unattended speakers (two-sample *t*-test, speaking rate: $t(40) = 0.934$, $P = 0.356$, two-tailed; disfluency: $t(40) = 1.598$; $P = 0.118$, two-tailed).

Coding of the communication behaviors

Verbal (e.g., turn-takings and interjections) and non-verbal responses (e.g., orofacial movements, facial expressions, and body gestures) during communications were coded by two coders. Results are summarized in Supplementary Table 1. An ANOVA on the frequency of responses showed significant main effects of number of speakers (F (1, 20) = 31.431, $P < 0.001$) and type of responses (F (1, 20) = 39.414, $P < 0.001$). A significant 2-way interaction was found between mode of communications and

number of speakers (F (1, 20) = 5.334, P = 0.032). No other significant 2-way interactions or 3-way interaction were found ($P > 0.05$). Further pairwise comparisons indicated that the frequency of non-verbal responses differed significantly between the face-to-face and back-to-back conditions (pairwise comparison, P = 0.017), but verbal responses did not (pairwise comparison, P = 0.59). These findings were consistent with the expectation that while verbal communications were shared between the face-to-face and back-to-back conditions, non-verbal communications were employed mainly in the face-to-face condition.

Exclusion of the multi-speaker tasks with freely chosen target speaker

This task was the same as the multi-speaker task with assigned target speaker except that the target speaker was not assigned *a priori*. Thus, the listener could choose the target speaker on her/his own, and she/he was allowed to switch attention freely and dynamically from one to the other.

The attended speaker was determined by two independent coders based on the video recordings. Time points where the listener attended to each speaker were marked. The criteria of attention were as follows: 1) direction of the listener’s face; 2) fixation at the target; 3) target of verbal and non-verbal responses. The inter-judge reliability (based on intra-class correlation (ICC)) for switching (vs. no switching) was computed at the time-point level for each individual group. The ICC was relatively high (i.e., from 0.833 to 1) in the face-to-face condition, but low (i.e., from 0.566 to 0.633) in the back-to-back condition. Although preliminary analyses of the data from this condition were consistent with our conclusions based on the other two tasks (details available from the authors), upon the recommendation of a reviewer, we refrained from directly comparing the multi-speaker tasks with freely chosen target to the other two types of tasks (i.e., the multi-speaker tasks with assigned target and the single-speaker tasks).

Supplementary Figures

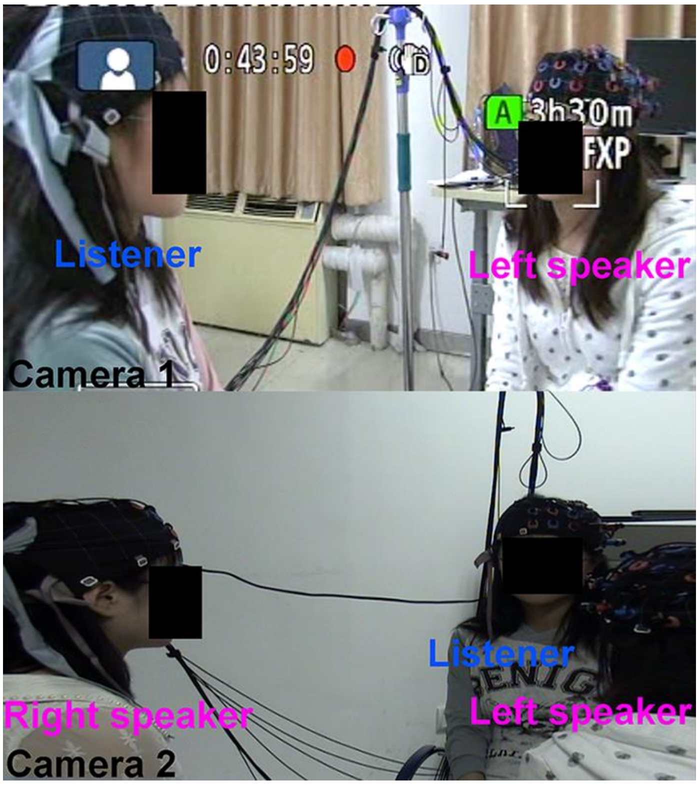

Supplementary Figure 1 The setup of the experiment. Shown are two sample frames from the face-to-face condition. Participants also performed the tasks in a back-to-back condition.

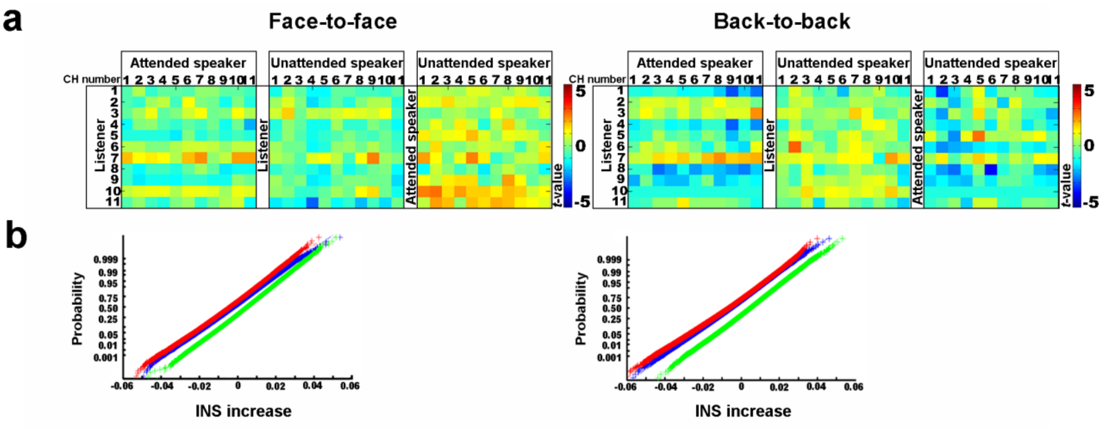

Supplementary Figure 2 Results of the permutation test. **a** Matrix of *t*-values for each task within each condition in a randomly selected test. None of CH combinations that reached significance in the original participant pairs reached significance in the random permutation test. **b** Results of 1000 permutation tests. The INS increase (i.e., task minus rest) for each CH combination are plotted together. The plots show normal distributions of the data.

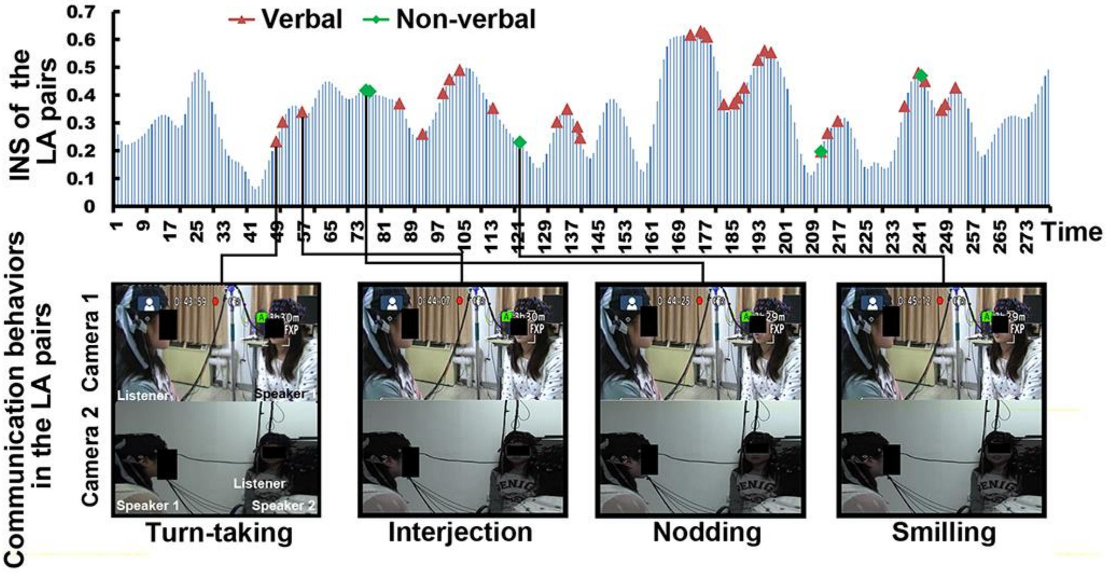

Supplementary Figure 3 The correspondence between INS and coded communication behaviors. A time course of INS for one randomly selected LA pair at TPJ-TPJ is shown. The corresponding communication behaviors were coded from video frames. Green points represent non-verbal communications, and red points represent verbal communications. The sections of the line without color points represent no communications.

Supplementary Table

Supplementary Table 1 Frequency of responses from the listener.

		Verbal	Non-verbal
Face-to-face	Single-speaker task	11%	6%
	Multi-speaker task	11%	5%
Back-to-back	Single-speaker task	8%	3%
	Multi-speaker task	9%	1%
